# Cell Signaling in Neuronal Stem Cells

**DOI:** 10.3390/cells7070075

**Published:** 2018-07-14

**Authors:** Elkin Navarro Quiroz, Roberto Navarro Quiroz, Mostapha Ahmad, Lorena Gomez Escorcia, Jose Luis Villarreal, Cecilia Fernandez Ponce, Gustavo Aroca Martinez

**Affiliations:** 1 Faculty of basic sciences and biomedical; Universidad Simón Bolívar, Barranquilla 080002, Colombia; mostapha.ahmad@unisimonbolivar.edu.co (M.A.); lorenagomez1212@gmail.com (L.G.E.); garoca1@unisimonbolivar.edu.co (G.A.M.); 2 School of Medicine, Universidad Rafael Nuñez, Cartagena 130001, Colombia; 3 Centro de Investigación en Salud para el Trópico, Universidad Cooperativa de Colombia, Santa Marta 470002, Colombia; robertcnavarro@gmail.com; 4 School of Medicine, Universidad Libre, Barranquilla 080002, Colombia; josevillarrealc@gmail.com; 5 School of Medicine, Universidad de Cadiz, Cadiz 110003, Spain; cecimat20@yahoo.com; 6 Clinica de la Costa, Barranquilla 080002, Colombia

**Keywords:** neural stem cell, Wnt/beta-Catenin, differentiation

## Abstract

The defining characteristic of neural stem cells (NSCs) is their ability to multiply through symmetric divisions and proliferation, and differentiation by asymmetric divisions, thus giving rise to different types of cells of the central nervous system (CNS). A strict temporal space control of the NSC differentiation is necessary, because its alterations are associated with neurological dysfunctions and, in some cases, death. This work reviews the current state of the molecular mechanisms that regulate the transcription in NSCs, organized according to whether the origin of the stimulus that triggers the molecular cascade in the CNS is internal (intrinsic factors) or whether it is the result of the microenvironment that surrounds the CNS (extrinsic factors).

## 1. Introduction

Neurodegenerative diseases are an important challenge from the point of view of public health, due to the increase in their prevalence, and because they have a significant impact on direct and indirect public expenditure for their treatment [1]. This has increased the interest in studying neural stem cells and the mechanism molecules involved in the process of neuronal differentiation. The search for therapeutic strategies for neurodegenerative diseases, elucidated several metabolic pathways such as the signal translation pathways of Sonic Hedgehog (Shh), Notch, Wnt, and Bone Morphogenetic Proteins (BMP), and the participation of some transcription factors such as Oct4, Sox2 and Nanog, which are responsible for regulating pluripotentiality in NSCs [2].

**Figure 1:**
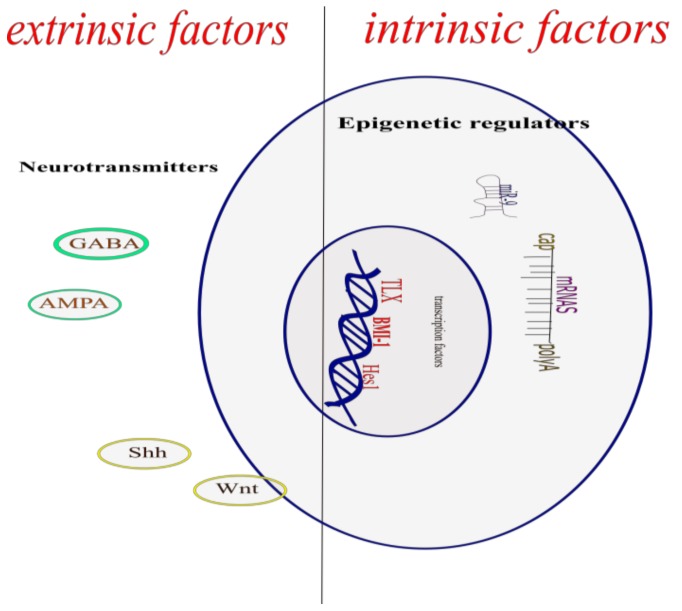
Molecular mechanism that regulates the differentiation of neuronal stem cells.

The process by which new neurons are generated is called neurogenesis; this involves multiple and complex pathways [3]. The NSCs give rise through asymmetric cell divisions, to the neural precursor cells which in turn by this same type of cell division, give rise to new functional neurons, both in the embryonic neural development and in the adult CNS. This creation of a new functional neuron includes the self-renewal of neural stem cells and neural precursor cells, the generation of neuroblasts that differentiate into young neurons that migrate, mature, and integrate into the pre-existing neuronal circuit, processes regulated by the dynamic interaction between the genome, epigenetic mechanisms, and extrinsic signals (Figure 1) [4].

This article reviews the molecular mechanisms involved in the process of differentiation of the NSCs.

## 2. Intrinsic Factors

### 2.1. Transcription Regulators

Transcription factors are protein complexes that participate in the regulation of the temporal space of genes, Which contribute to the control of gene expression variations in NSCs, at a determined time, Interestingly, a great variety of these complexes have been found regulating NCSs final cellular phenotype. Among these transcription factors, Tlx orphan nuclear receptor is essential for the maintenance and self-renewal of NSCs in adult brains [5], Tlx gene is expressed in sensory neurons, as well as postsynaptic neurons in the central relay stations. In addition, expression of Tlx3 with two other transcription factors, Phox2b and DRG11, differentiates somatic circuits (Tlx3 + DRG11 +) from visceral sensory circuits (Tlx3 + Phox2b +). Therefore, Tlx expression determines neuronal connectivity. Within sensory relay stations, Tlx genes establish excitation on the inhibitory transmitter phenotype [6,7]. Tlx is found in the neurogenic regions of the retina, telencephalon, nasal placode, and diencephalon [8]. TLX is distributed through the cortex, showing an strong but dispersed expression in the subgranular zone (SGZ) of the dentate gyrus (GD), and grouped expression in the subventricular zone (SVZ) of the lateral ventricle [9]. The main function of TLX in the adult brain is to prevent NSCs early differentiation by controlling the expression of an extensive gene network. In this way, TLX keeps NSCs in an undifferentiated and self-renewing state, specifically, modulating the signaling of p53 pathway [10].

On the other hand, TLX-null cells isolated from TLX-null mice brains do not proliferate. Moreover, reintroduction of TLX into TLX-null cells rescues its ability to proliferation and self-renewal [5]. In vivo, TLX mutant mice show a loss of cellular proliferation and reduced neural precursors in the neurogenic areas of adults brains. TLX represses the expression of markers of astrocytes, such as GFAP (acidic protein fibrilar glial), and the tumor suppressor gene, pten (phosphatase and tensin homolog) in NSCs, suggesting that transcriptional repression is crucial to maintain the undifferentiated state of these cells [5,11]. In the proliferative state, Tlx cooperates with HDAC (ASSOCIATED FACTOR COMPLEX) to inhibit the transcription of miR-9 in NSCs, In the differentiation state, miR-9 inhibits the expression of Tlx and promotes ongoing neuronal differentiation [12]. miR-9 and TLX form a feedback regulatory loop to coordinate the proliferation and differentiation of retinal progenitors [13]. The elucidation of the TLX-regulated network to produce these results would be a significant advance in the understanding of the self-renewal and neurogenesis of NSCs.

### 2.2. Estrogen Receptors

Estrogen receptors (ERs) are part of the family of NR3A nuclear receptors and also known as steroid hormone receptors. The estrogen receptor subtypes ER alpha (NR3A1) and ER beta (NR3A2) are expressed in the nucleus, cytoplasm and membrane [14]. It has been shown that the activation of estrogen receptors by 17 beta estradiol (E2) regulates the proliferation of embryonic NSCs mediated by overexpression of the cyclin-dependent kinase inhibitor, p21^Cip1^ [15], while promoting the proliferation and differentiation to glial cells of NSC embryonic rat in the absence of mitogens Epidermal growth factor (EGF), Fibroblast growth factor-2 (FGF-2) or differentiation factors [16]. In human embryonic NSCs, E2 acts through ERs to induce its differentiation into dopaminergic neurons [17]. Estrogen receptors are involved in the induction of adult neurogenesis by interaction with insulin-like growth factor (IGF-1), because E2 and IGF-1 have a synergistic effect to promote neurogenesis [18].

### 2.3. Complex Protein Polycomb BMI-1

Bmi1 is an epigenetic regulator that belongs to the polycomb group complex, which participates in the transcriptional control of genes, through the ubiquitination of histone H2A Lys-119 [19], silencing the expression of genes [20,21]. Bmi-1 has been shown to be necessary for the postnatal maintenance of NSCs in the central nervous system [22]. Deficiency in Bmi1 leads to progressive delay of postnatal growth and neurological defects [23]. Mice in which the Bmi1 gene is silenced exhibit a defect of postnatal self-renewal that leads to the depletion of adult stem cells [22]. One way Bmi1 promotes maintenance of adult stem cells is repressing inhibitors of cyclin dependent kinases, p16 Ink4a and p19 Arf [24].

In addition, Bmi-1 represses transcription at the Ink4a-Arf locus [25], encodes two inhibitors of cell proliferation [26], Ink4a encodes p16 Ink4a, an inhibitor of cyclin-dependent kinase that promotes activation of Rb, the Arf encodes p19 Arf, which promotes the activation of p53, p16 Ink4a, and p19 Arf induced in cultured primary cells and that these cells can grow old [27], overexpression Bmi-1 can prevent senescence and prolong the replicative lifespan of primary cell to reduce expression of p16 Ink4a and p19 Arf [28], the elimination of Ink4a-Arf from mice Bmi-1-/-rescues the ability of mouse embryonic fibroblasts to proliferate in culture and at least partially recover the defects in the development of the cerebellum [25], p16 The expression of Ink4a is elevated in Bmi-1-/-neural stem cells and the elimination of Ink4a from Bmi-1-/-mice partially rescues the self-renewal of neural stem cells in culture [22].

### 2.4. The Sox Family

The Sox protein family are characterized belongs to the Sry family of genes and contains a DNA binding domain called the high mobility group domain (HMG), with a high homology with the same Sry-box, of the HMG-box of the Sry protein. The DNA-binding domain consists of three alpha helices of the HMG box occurring specifically in the minor groove and induces a strong curve, thus changing the architecture of DNA. These conformational changes allow interactions with other transcription factors. These changes in the transcription factors with which the members of this family interact within a wide range of cellular contexts, induce diverse effects on the cellular metabolism [29].

Sox genes, plays a role in maintaining the undifferentiated state of NSCs in a context-dependent manner; in vertebrates, SoxB1 (Sox1, Sox2, and Sox3) factors are widely expressed in the proliferation state of neuronal stem/progenitor cells, during the development, as well as in adulthood [30]. Its expression is located later in the ventricular layer in the neuronal cortex, where the NSCs and their precursor cells are present after the middle fetal period. During this period, Sox2 is not expressed in layers where differentiated neurons are present. Moreover, It has shown that SoxB1 factors play a role in maintaining the undifferentiated state of embryonic neural progenitors [31].

The overexpression of Sox2 and Sox3 inhibits the neuronal differentiation of neuronal progenitors, on the contrary, the expression of a dominant negative form of Sox2 and Sox3 results in the premature departure of neuronal progenitors from the neurons, cell cycle and the onset of neuronal differentiation [30], in addition to its function in early brain development [31].

Sox2 is also necessary for maintaining neural stem cells in neurogenic areas in adults. Regulatory mutations of Sox2 cause neurodegeneration and impaired adult neurogenesis [32]. In parallel, Sox10, a member of the subfamily Sox E, maintains multipotence of stem cells from the neural crest in the peripheral nervous system [33]. Based on these findings, it is conceivable that the feedback loop mechanism centered on Sox2 that involves the Sox2 target genes serves as an important system for the self-renewal mechanisms of the NSC. In fact, several combinations of transcription factors have been used to generate induced pluripotent stem cells (iPSCs), all of which include Sox2 [34].

### 2.5. Multiple Genes Helix-Loop-Basic Helix (bHLH)

A basic helix-loop-helix (bHLH) is a structural motif that is characterized by two α-helix connected by a loop. In general, the transcription factors that include this domain are dimeric, each with a helix containing basic amino acid residues that facilitate DNA binding [35]. The genes helix-loop-basic helix (bHLH) also plays a critical role in the regulation of the maintenance and differentiation of neural stem cells [36,37]. The Hes genes, hairy Drosophila homologs and excision enhancer, are genes of bHLH repressor type, among which seven members of the Hes family. Hes1 and Hes5 are essential effectors of Notch signaling, whose expression is regulated positively by the activation of Notch [38]. Hes1 and Hes5 are highly expressed by NSCs [39] and misexpression, and inhibit neuronal differentiation and maintain NSCs in the embryonic brain [40]. An example of this is a study in which neural progenitors were subjected to differentiation by premature neuronal ions; after this treatment, mice were identified to have silenced Hes1 and Hes5 [41], suggesting that Hes1 and Hes5 are essential for NSC maintenance and self-renewal.

Additionally, bHLH genes related to Hes, Hesr1, and Hesr2 are also expressed by NSCs in embryonic brains and act as Notch signaling effectors, and also regulate NSC maintenance and self-renewal, possibly through cooperative action with the Hes [37]. These data suggest that intrinsic transcription factors can work together to coordinate NSC maintenance and renewal.

Ascl1 (Mash1) is a member of the basic family of helix-loop-helix (bHLH) transcription factors involved in the sequential targeting of NSC during embryonic and adult neurogenesis [36]. By using lineage tracing in vivo with (photo-activatable Cre recombinase PA-Cre) CRE-inducible recombinase, it has been discovered that the expression of Ascl1 in the adult SVZ is restricted to differentiated transit amplifying cells in GABAergic interneurons in the olfactory bulb [42]. In the adult hippocampus, Ascl1 transiently expresses the type 2 progenitor cells that subsequently develop in glutamatergic neurons as granular cells [42]. Interestingly, asv1 retrovirus-mediated overexpression in vivo instructed adult hippocampal progenitor cells to generate cells of the oligodendrocytic lineage instead of generating excitatory granitic cells, the predominant phenotype generated under physiological conditions [43].

### 2.6. The cAMP Response Element Binding Protein (CREB)

Binding protein response element cyclic adenosine monophosphate (cAMP) (CREB) is a transcriptional factor l which mediates process growth and cell development, and is the common phosphorylation substrate in several routes mediated by signaling kinase, including cAMP/protein via kinase A, calcium calmodulin, and NMDA receptor, as well as MAP kinase signaling induced by neurotrophins through Trk receptors [44], Upon phosphorylation, CREB form dimers and binds to response elements cAMP within the promoter regions of target genes [45]; more specifically, phosphorylation mediated cAMP CREB increases neurogenesis to stimulate proliferation progenitor cell and regulates the survival of newborn neurons in the adult hippocampus in vivo [46], In addition, CREB’s autonomous cellular inactivation through retrovirus-mediated overexpression of dominant negative CREB in newly-born adult mouse hippocampal cells is characterized by altered differentiation and morphological maturation of granular cells. of newborns [47]. Similarly, in signaling CREB-autonomous cells, it is critically involved in regulating survival, migration, and morphological differentiation of cough Neuroblastoma in adult SVZ [48]. Curiously, loss of CREB increases Pax6 expression, suggesting that the effects of CREB signaling on the survival of immature neurons in rostral migratory flow (RMS) may be mediated by Pax6 modulation [49].

### 2.7. Pax6

Paired box protein (Pax6) is a transcription factor crucial to model the telencephalon during development [50], and the adult subventricular zone SVZ, Pax6 is expressed in immature neurons migrate into the RMS to the olfactory bulb [51], Retroviral overexpression of Pax6 in neurosphere cultures derived from adult telencephalon, which has been shown to be composed almost entirely of cells derived from the neurosphere towards a neuronal fate [52], in addition, the in vivo analysis revealed that Pax6 restricts the precursor cells in the RMS to a neuronal destination, and it is enough to induce neuroblast differentiation to dopaminergic postmitotic periglomerular neurons [53].

### 2.8. Dlx2

Distal-minus 2 (Dlx 2) is a transcription factor of a homeobox that is expressed by transit amplifying cells and migrating neuroblasts in adult SVZ [54]. In a study by Brill et al., overexpression of Dlx2 produced a significant increase in neuronal differentiation and the migration rate of the neuroblasts that migrated to the olfactory bulb. More specifically, Dlx2 promoted the differentiation of neuronal precursors into dopaminergic neurons periglomerular to olfactory bulb, in cooperation with Pax6 [55]. This study also demonstrates that Dlx2 is required to maintain the proliferation of SVZ precursors. Inhibition of Dlx2 expression in the adult DG did not affect hippocampal neurogenesis, suggesting a region specificity of the regulation of L to neurogenesis by Dlx2 [55], Subsequent in vitro studies have shown that Dlx2 promotes lineage progression from the stem cells to the transient amplifying cells by increasing the expression of the EGF receptor in the SVZ stem cells [56]

### 2.9. Emx2

Emx2 is a homeobox transcription factor that is widely expressed in the developing brain, and is important for proper morphogenesis of the CNS [57,58]; in the adult brain, Emx2 is expressed in SVZ precursors, as well as in DG of the hippocampus [59]. It was shown that Emx2 negatively regulates the proliferation of SVZ progenitor cells promoting the symmetric division of stem cells, generating a more differentiated than undifferentiated progeny [59], and this study also explains why the overexpression of Emx2 resulted in a decrease in the proliferation of NSC clones derived from SVZ, by promoting asymmetric cell divisions [60].

### 2.10. Tbr2

Tbr2 is a transcription factor of T-box expressed in neurogenic regions of the adult and developing brain [61,62], and the adult DG-expressed TBR2 in intermediate neuronal cell progenitors [62], and silencing TBR2 resulted in neurogenesis altered DG in vivo due to the failure of the NSCs to differentiate into neuroblasts posmitotically [60].

By organizing the transcriptional factors into the section “Intrinsic Factors”, the authors detailed the most important factors responsible for the self-renewal, proliferation, and specification of neuronal stem cells. The missing connection between these factors is that of the well-studied cooperative function and crosstalk during neural reprogramming, which should not be ignored in these years. For example, the increased induction of neuronal cells from stem cells or fibroblasts was generated by the overexpression of genes of the bHLH family and the POU4F2 family. Ascl1 and Dlx2 worked together to induce the generation of GABAergic neurons from human stem cells.

### 2.11. Master Regulators

The process of differentiation to neurons of the stem cells implies a coordinated response by inhibiting or activating the previously described transcription factors, knowledge that has been achieved mainly through studies of reprogramming of somatic cells, in which the forced expression of transcription factors induces a neuronal fate in human pluripotent cells. Among the transcription factors, we highlight Sox proteins that participate in various processes in the CNS, among these the events of cell determination, lineage progression, differentiation, cell survival, and phenotypic homeostasis. These functions are due to their role as classical transcription factors that induce the transcription of specific effector genes of the cell type and stage, through the interaction with other transcription factors, and their capacity to recruit chromatin modifiers. Its impact on microRNA expression in addition, allows them to influence and perfect cell development and identity, not only at the epigenetic and transcriptional levels, but also at the post-transcriptional level [29]. Likewise, from knowledge about the transcriptional factors, Sox2 has been permitted to induce a neuronal phenotype in progenitors, as well as being the target of many genes, allowing an important system for the self-renewal mechanisms of the NSC. This means that the overexpression of Sox2 and Sox3 inhibits the neuronal differentiation of the neuronal progenitors and causes them to retain undifferentiated properties; on the contrary, the expression of a dominant negative form of Sox2 and Sox3 results in the premature departure of the neuronal progenitors from the neurons’ cell cycle, allowing the onset of neuronal differentiation [30].

Together with Sox2 other TF, such as OCT4 and NANOG play determining roles in the maintenance of pluripotency. In mouse embryos that do not express Oct4, the pluripotent internal cell mass is not generated and therefore cannot survive beyond the blastocyst stage [63]. The non-expression of Nanog induces in mouse embryos the non-development of epiblast [64]. Additionally, these transcription factors regulate in a redundant manner a large part of their target genes that form a characteristic network that induce cellular pluripotency. Its ability to reprogram somatic cells is convincing proof of its role as a key regulator of pluripotency [65].

On the other hand, the coupling of several of these transcription factors involved in cellular difference allow a neural phenotype, which can be drawn as follows, To initiate, the TLX transcription factor mediates the expression control of an extensive gene network in the adult brain to prevent early differentiation of NSCs, allowing the maintenance and self-renewal of NSCs in the adult brains. Likewise, Dlx2 promoted the differentiation of neuronal precursors into dopaminergic neurons periglomerular in the olfactory bulb in cooperation with Pax6; in addition, Pax6 restricts the precursor cells in the RMS to a neuronal destination, and this is sufficient to instruct induce neuroblast the differentiation of neuroblasts to dopaminergic postmitotic periglomerular, or neuronal dopaminergic postmitotic cells [66]. Similarly, signaling of CREB-autonomous cells is critically involved in regulating survival, migration, and morphological differentiation of cough neuroblastomas in adult SVZ, but loss of CREB induces an increase in the signal for Pax6 expression, suggesting that the effects of CREB signaling on the survival of immature neurons in RMS may be mediated by Pax6 modulation [48]. In other places, Hes1 and Hes5 are highly expressed by neural stem cells or misexpression, and inhibit neuronal differentiation and maintain neural stem cells in the embryonic brain [40].

## 3. Epigenetic Regulators

Epigenetic mechanisms, including a broad set of DNA methylation and histone modification, have recently emerged as an important link between external environmental control and the transcriptional control of gene expression in NSCs [67]. Epigenetic modification is are defined as stable and hereditary changes in gene expression patterns that are not encoded in the primary DNA sequence itself, resulting in new cellular phenotypes without altering the real genomic sequence [68]. DNA methylation occurs predominantly in the cytosine residues of dinucleotides CpG to generate 5-methylcytosine in the pyrimidine ring, and the methylation status of the DNA plays critical roles in the regulation of gene expression during development [69]. Methylation of CpG sites regulates expression by blocking DNA binding or the binding of methyl CpG binding proteins (MBD). The binding of MBD to methyl-CpGs, in turn, results in the repression of gene transcription by the recruitment of histone deacetylase repressor complexes and the subsequent deacetylation of histones [70].

Protein of the methyl-CpG binding domain (MBD1) is expressed as the highest concentration in the adult hippocampus [71]. Neurogenesis hippocampal and impaired spatial learning capacity [71]. Subsequently, it was demonstrated that MBD1 facilitates neuronal differentiation by directly joining the mitogen promoter NSC FGF-2. The MBD1-induced methylation of the FGF-2 promoter results in the down-regulation of FGF-2 expression, thus allowing adult hippocampal progenitor cells to undergo neuronal differentiation [72]. In addition, it has recently been shown that MBD1 promotes neuronal differentiation by decreasing miR-184 microRNA expression [73]. Interestingly, miR-184 promotes the proliferation of adult NSCs by down-regulating Numbl, a protein that was previously shown to be important for cortical brain development; therefore, MBD1 promotes neurogenesis in the adult brain through Numbl and requires the deletion of miR-184 [74].

The CpG-binding protein of methyl (MeCP2) is another MBD that is predominantly expressed in neurons in the mammalian CNS, and participates in the regulation of neurogenesis in the adult [75,76]. Newborn neurons in the DG of mice deficient in MeCP2 show profound deficits in neuronal maturation and spine formation [77]. MeCP2 is important for maintaining neuronal identity and phenotypic characteristics by promoting methylation of the GFAP promoter near the initiation site in neuronal precursors, thus suppressing the expression of GFAP in developing neurons [78]. On the other hand, overexpression of MeCP2 was shown to inhibit astroglia and promote neuronal differentiation in embryonic cells NSCs in vitro and in vivo [79]. Although MeCP2 was formerly thought to be involved in the regulation of neuronal maturation rather than the choice of the fate of progenitors in the adult brain, a recent study demonstrated a role for MeCP2 in the regulation of proliferation and differentiation of neuronal stem cells into through miR-137 microRNA. On the other hand, MeCP2 in cooperation with Sox2 represses the expression of miR-137, thus promoting NSC differentiation in adults [80].

Growth arrest and protein-induced damage to DNA beta (GADD45b), a protein previously implicated in DNA demethylation, has recently been identified as an important regulator of activity-induced neurogenesis [81]. GADD45B is expressed in adult DG and expression is increased temporarily in response to electroconvulsive activity. GADD45b mediates the proliferation of adult hippocampal progenitor cells induced by the activity and dendritic growth of newborn neurons, promoting demethylation and thus the induction of target gene promoters critical for adult neurogenesis, including BDNF and FGF [82].

TET1 is a member of the TET family of proteins, a group of Fe(II)/2-oxoglutarate-dependent dioxygenases that hydroxylates the 5-methyl group of the cytosine ring to produce 5-hydroxymethylcytosine [83,84]. The TET1-mediated conversion of 5-methylcytosine to 5-hydroxymethylcytosine is important for the demethylation of DNA in mammalian cells [85]. Interestingly, the demethylation induced by neuronal activity of the brain-derived neurotrophic factor (BDNF) and FGF promoters was completely abolished in the adult DG after the overexpression of short hairpin RNA (shRNA) against TET1 in vivo, suggesting an important role for TET1 in the regulation of neurogenesis induced by activity in the adult hippocampus [86].

Histone methyltransferase of mixed lineage 1 leukemia (Mll1) is expressed in both embryonic and adult SVZ, and critically regulates the expression of numerous developmental genes [87]. Transgenic mice deficient in Mll1 exhibit proliferation of intact neural stem cells, survival, and glial differentiation, but show a severely affected neuronal differentiation in adult SVZ. In addition, Mll1-dependent neuronal differentiation of adult SVZ NSCs requires direct interaction and adequate transcriptional activation of the Sox2 gene [88].

Family members of fragile X mental retardation proteins, including FMRP, FXR1 and FXR2, regulate messenger RNA (mRNA) translation by direct and selective binding to RNA, and association with polyribosomes [82,89]. The loss of functional FMRP in NSCs in adult DG in vivo resulted in greater differentiation of astrocytes at the expense of neuronal differentiation and severe defects in hippocampal dependent learning, suggesting an important regulatory role for FMRP in neurogenesis and adult learning [90]. In addition, FMRP-induced neurogenesis requires intact Wnt signaling, as well as the presence of the downstream mediator Neurogenin1, a transcription factor bHLH [91]. In the adult olfactory bulb, FMRP is required for autonomous neuronal cell differentiation by regulating dendritic spine production and morphogenesis [92]. FXR2 regulates hippocampal but not SVZ neurogenesis by facilitating BMP signaling, FXR2 represses noggin expression by reducing the stability of noggin mRNA, allowing the proliferation and differentiation of NSCs mediated by BMP [93].

## 4. Regulation of microRNAs

Association between microRNAs (miRNAs) and the nuclear receptor TLX in the self-renewal of neural stem cells and the determination of cell fate.

Recent studies revealed that the interaction between the miRNAs and the nuclear receptor TLX plays an important role in the specification and determination of the fate of neural stem cells [94]. 

In relation to the above, it has been demonstrated that TLX is an essential regulator of the self-renewal of neural stem cells, through regulatory genes that are important in controlling the proliferation of stem cells [5]. An example of the above is miR-9, a miRNA specific to the brain that is expressed in the neurogenic areas of embryonic and adult brains [95]. Interestingly, miR-9 and TLX form a feedback regulatory circuit, which in turn regulates the proliferation and differentiation of neural stem cells [12]. TLX is highly expressed in neural stem cells, whose expression is reduced after differentiation. On the other hand, the expression of miR-9 increases during differentiation. The temporal relationship between miR-9 and TLX expression supports the hypothesis that miRNAs induced during the differentiation of neural stem cells ensure the transition of cell fate by suppressing essential factors of stem cell maintenance. MiR-9 functions in neural stem cells through TLX directed expression. Interestingly, TLX also acts as a transcriptional repressor of miR-9 genes [12]. This cycle of negative feedback between TLX and miR-9 would allow a rapid transition from neural stem cells to differentiated cells.

In addition to TLX, other target genes have been predicted and tested for miR-9, including those encoding the transcription factors REST, FoxG1, Senseless, Hairy/E transcription factors (spl) Her 5, Her 9 and the components of the FGF signaling pathway, the DL DLO transcription factor of Drosophila, and statin, a protein that increases microtubule instability [96]. These studies raise an interesting possibility that miR-9 may act as a regulatory switch in different stages of development and in different organisms when interacting with the target mRNAs differentially expressed in a specific temporal and cellular context [97]. The regulatory cycle between miRNAs and transcription factors can serve as a general mechanism to control the balance between proliferation and differentiation of stem cells, and to allow the determination of cell fate in a timely manner.

Recently it was shown that miRNA lethal-7b (let-7b) also regulates the decision of the fate of neural stem cells [98]. Overexpression of let-7b led to the inhibition of proliferation of neural stem cells and accelerated neuronal differentiation, whereas the antisense drop of let-7b resulted in an increased proliferation of neural stem cells. In addition, in utero electroporation of let-7b to mouse embryo brains led to a reduction in cell cycle progression in neural stem cells. Interestingly, this study provided a direct link between let-7b and the nuclear receptor TLX. let-7b regulates the proliferation and differentiation of neuronal stem cells by targeting TLX and its subsequent effector, cyclin D1. The characterization of the let-7b-nuclear receptor signaling cascade provides a new understanding of the role of let-7b in determining the fate of neural stem cells. Let-7b has also been shown to target Hmga2 to reduce the self-renewal of neural stem cells in the brain of the elderly [99]. Together with our observations on the role of let-7b in adult and embryonic neural stem cells through TLX and cyclin D1, these studies suggest that let-7b works in neural stem cells in a spectrum of stages of development through different key molecules.

### Disturbance between miRNA and Epigenetic Regulation in Neural Stem Cells and Neurogenesis

It is known that epigenetic mechanisms, including DNA methylation and histone modification, play an important role in the modulation of stem cell proliferation and differentiation [100]. DNA methylation represses gene transcription by directly blocking the access of transcription factors to their binding sites or by indirectly recruiting methyl-CpG-binding proteins (MBD or proteins from the Kaiso family [101], including MBD1, MBD2, MBD3, MBD4, and MECP2, the importance of epigenetic regulation in brain development and neurological disorders has been well documented. The mutations of Novo in MeCP2 leads to Rett syndrome, a dominant neurodevelopmental disorder linked to the X chromosome [102], and MBD1 mutations have been found in a subset of autistic patients [103]. MBD1 deficiency in mice results in impaired adult neurogenesis and hippocampal dependent learning [71]. It has been shown that both MBD1 and MeCP2 regulate the proliferation and differentiation of neural stem cells or the maturation of young neurons. However, the identification of their downstream targets by classical analyses of gene expression have yielded limited results [104].

It has been described that the MeCP2 regulates specific miRNAs in neural stem mouse cells [80], they have shown that one of these miRNAs, miR-137, promotes proliferation and suppresses the differentiation of neural stem cells by repressing translational of Ezh2, a histone H3 lysine 27 methyltransferase and a member of the Polycomb group (PcG) protein family [105]. On the other hand, the expression of miR-137 is also positively regulated in melanoma neurons deficient in MeCP2. miR-137 has a significant impact on the dendritic morphogenesis of young hippocampal neurons. Interestingly, the neuronal maturation function of miR-137 is achieved by the translational repression of Mind bomb-1, a ubiquitin ligase known to be important for neurogenesis and neurodevelopment [106], rather than Ezh2. Therefore, miR-137 can have multiple functions during different stages of neurogenesis.

In a parallel experiment, it is discovered that MBD1 regulates the expression of a subset of miRNAs in adult neural stem cells and one of them is miR-184, a printed miRNA whose genomic region has been shown to be bound to MeCP2 in neurons [107]. We show that in adult neural stem cells, miR-184 is regulated by MBD1, but not by MeCP2; on the other hand, miR-184 promotes the proliferation of neural stem cells and inhibits differentiation by targeting Numblike [73]. Therefore, MBD1, miR-184, and Numblike can form a regulatory network that controls the balance between proliferation and differentiation of neural stem cells.

Although is likely that the lack of coordination between miRNA and DNA methylation is a common mechanism that regulates critical cellular processes, the evidence for this concept in mammalian neural stem cells is limited. Our results showed that perturbation between epigenetic regulation and the miRNA pathway could play an important role in the modulation of adult neurogenesis.

## 5. Extrinsic Factors

### 5.1. Metabolic Pathways Associated Neurodiferencioin: Via Wnt/Beta-Catenin

The Wnt signaling pathway is a highly conserved signaling pathway that has been implicated in the development of the nervous system, including neural tube formation, the development of dorsal root ganglia, and the development of the midbrain [108]; interruption of the signaling pathway Wnt has been associated with several pathologies of the CNS, such as schizophrenia, mood disorder, autism, and Alzheimer’s disease [109].

The Wnt ligands constitute a family of glycoproteins secreted by autocrine and paracrine pathways that are involved in various cellular processes of various development [110]; in the absence of the Wnt ligand, a key modulator of the Wnt pathway is activated by glycogen synthetase kinase-3 beta (GSK-3beta) to form a degradation complex, consisting of: axons and proteins that contains beta transductor repeats (beta-TrCP), resulting in phosphorylation and ubiquitination of beta-catenin and the subsequent degradation of beta-catenin by the proteasome [111]. The continuous degradation of beta-catenin in the absence of Wnt maintains a low level of intracellular beta-catenin. The purpose of Wnt is sequestration and the continuous degradation of beta-catenin in the absence of cell factor T (TFA) and subsequent binding factor T/enhancer binding factor lymphoid (TCF/LEF) lymphocytes; the ligand Wnt prevents transcription of the Wnt target genes. However, in the presence of an extracellular ligand, Wnt and its Frizzled receptor form a ternary complex with the co-receptor of the protein related to the low-density lipoprotein receptor 5/6 (LRP5/6) [112]. For GSK-3beta of the degradation complex, in the absence of ongoing beta-catenin degradation, stabilized beta-catenin enters the nucleus and is associated with TCF/LEF transcription factors, resulting in the transcription of object genes from Wnt [113].

Several studies have addressed the role of Wnt signaling in neurogenesis; recently it has been shown that Wnt3 is expressed in cells of the DG and cultured astrocytes of hippocampus, and that the signaling of GSK3beta/beta-catenin is active in the SGZ and in the dentate granular cell layer [114]; the same study showed that Wnt signaling derived from astrocytes mediates neuroblast proliferation and neuronal differentiation in hippocampal progenitor cells derived through the beta-catenin pathway. Similarly, the injection of lentiviruses expressing dominant negative Wnt in adult DG resulted in a marked reduction in neurogenesis compared to wild-type Wnt, suggesting an important role for Wnt signaling in adult hippocampal neurogenesis in vivo [114]. NeuroD1 is a basic transcription factor helix-loop-helix (bHLH) with pro-neurogenic functions as a downstream mediator of Wnt-induced neurogenesis from adult hippocampal neural progenitors [115].

Interestingly, NeuroD1 expressed in dividing neural progenitors and immature granule neurons in DG adults but not in hippocampal neural progenitors expressing Sox2; however, in the presence of Wnt, extracellular beta-catenin is accumulated in the activating complex that is formed in the nucleus with TCF/LEF, resulting in the transcriptional activation of NeuroD1 and subsequent neuronal differentiation [115], in mice in which NeuroD1 was silenced, showing the need for NeuroD1 for neurogenesis in the adult hippocampus in vivo by facilitating the survival and maturation of adult neurons [116]. Despite this, convincing evidence of the role of Wnt/beta-catenin in the regulation of neurogenesis by neuronal differentiation has much to be clarified; a recent study by Mao et al. demonstrated that activation of the Wnt/beta-catenin pathway promotes proliferation rather than differentiation of adult NSCs [117].

### 5.2. The Signaling Pathway of Notch

Notch signaling affects a wide range of cellular processes in the developing nervous system, including cell proliferation, differentiation, and apoptosis [118,119], Notch receptors are transmembrane single-pass heterodimers that are activated by forming a complex of ligands bound to the membrane in the neighboring cell, binding of the ligand Delta-1 and Jagged-1 results in gamma-secretase-mediated cleavage of the transmembrane domain, and subsequent release of the intracellular Notch domain (NICD) in the cytosol. The NICD then translocates to the nucleus where it forms a complex with the DNA-binding protein RBPj; on the other hand, the NICD-RBPj complex in turn acts as a transcriptional activator and inducer of the expression of transcription factors bHLH, the excision enhancer (HES), and others [120].

Signaling by Notch in neurogenesis has also not been demonstrated to be a common feature of NSCs in the nervous system of adults. It is expressed in area subventricular (SVZ) and cortical gray matter (SGC) from mammalian brain adults [121]. Notch regulates the maintenance of adult NSCs, promoting the exit of the cell cycle and the group of adult neuronal progenitors [122]. The conditional deactivation of RBPj in the adult SVZ leads to the differentiation of all types of B cells into neurons that amplify the transit, which results in depletion of the set of neural stem cells and the subsequent premature cessation of neurogenesis [123].

Similarly, it was found that the Notch pathway was necessary for the expansion and self-renewal of Nestin-expressing cells in the hippocampus [124]. In addition, Ehm et al. showed that conditional inactivation of RBP resulted in an initial increase in the neurogenesis in the hippocampus due to premature neuronal differentiation of Sox2-positive progenitors, this in turn resulted in the subsequent depletion of a group of neural stem cells which are Sox2-positive, and the eventual suppression of neurogenesis in the adult hippocampus, indicating an important role for Notch signage in the maintenance of adult NSCs [125].

Therefore, Notch signaling seems to be involved in the regulation of the identity and plasticity of the niche cells, EphB2 acts as a mediator downstream of Notch signaling and prevents the differentiation of ependymal cells in niche of astrocytes in the adult SVZ [126], EGF, and Notch receptor signaling in the maintenance of neuronal stem and progenitor cells in the adult SVZ (subventricular zone). The signaling of the EGF receptor in transit amplification (type C cells) did not autonomously inhibit the proliferation and cellular self-renewal of type B cells by suppressing Notch signaling in type B cells in the adult SVZ [127].

Interestingly, EGF receptor signaling suppressed Notch signaling by promoting the ubiquitination of Notch 1 and degradation through Numb induction, a degradation of the emerging Notch receptor [128], therefore Notch signaling is required to maintain a reservoir of undifferentiated cells to ensure ongoing neurogenesis during adult life [129].

### 5.3. Sonic Hedgehog Path

Sonic hedgehog (Shh) is a soluble extracellular signaling protein that plays a role in cell differentiation in the neural tube and limb bud [130]. It has been found that Shh signaling is critical in several processes during the development of the nervous system, such as neuronal differentiation of the ventral forebrain, dopaminergic differentiation of the mesencephalon, and proliferation of neuronal precursors of the cerebellum [131,132]. The Shh pathway mediates its action through a complex receptor of the patchy transmembrane receptor protein (Ptc) and its co-receptor coupled to the G protein Smoothened (Smo), which are located preferentially in the primary cilia [133]; in the absence of the ligand, Shh and Ptc repress the signal transduction of the receptor Smo, thereby inhibiting the transcription of the target gene Shh [134,135]. 

More recently, it has been shown that Shh plays a role in neurogenesis in the brain of an adult mammal, The Shh, Ptc, and Smo receptors are expressed in the adult hippocampus and in the progenitors derived from this region [136,137]. In addition, several components of the Shh signaling cascade are expressed early in the postnatal period in the SVZ, as well as in the adult SVZ [138,139]. The overexpression of Shh in the adult hippocampus through an adeno-associated viral vector administered to the DG resulted in a significant increase in proliferation of hippocampal progenitor cells in vivo [137].

In contrast, cyclopamine, an inhibitor of Shh signaling, reduced granule cell proliferation in the adult DG when it was administered directly into the adult hippocampus [137] and the lateral ventricle [140], in conditional inhibition of signaling through the mediator shh, downstream Smo demonstrated a significant reduction in proliferation of progenitor cells in postnatal hippocampal and SVZ [141]. Moreover, Smo in the neural precursor cells shows defective hippocampal neurogenesis, as indicated by a small DG, and a marked reduction in the proliferation of neural stem cells in the adult dentate gyrus in vivo [142].

In addition, Shh is an important regulator of cell migration in the brain of adult mammals, several studies have addressed the role of shh in neuronal migration in adult SVZ by means of conditional de-activation of Smo as well as adenoviral overexpression of Hip, a negative regulator of the Shh pathway, Interestingly, the loss of Shh signaling in the adult SVZ resulted in the autonomous non-cellular failure of the neuroblasts (type A cells) to migrate to the olfactory bulb via the RMS pathway [143].

## 6. Growth Factors and Neurotrophic Factors

Neurotrophic factors are extracellular signaling proteins in the developing central nervous system, and in adults in mammals, four neurotrophic factors have been identified: nerve growth factor (NGF), BDNF, neurotrophin 3 (NT-3), and neurotrophin 4/5 (NT-4/5) [144,145]. Neurotrophins bind to tyrosine kinase receptors known as Trk receptors and their co-receptor p75NTR. There are three different Trk receptors, namely TrkA, TrkB, and TrC, which have different preferential binding affinities for different neurotrophins [146,147].

While NGF binds preferentially to TrkA, BDNF and NT-4/5 to TrkB, and NT-3 to TrkC [148,149], the four neurotrophic factors bind to p75NTR at the cell surface, which serves to increase and facilitate the binding of the respective neurotrophic to its specific Trk receptor, the binding of the ligand induces the dimerization of Trk receptors and their autophosphorylation at specific tyrosine residues in the cytoplasmic domain, which leads to the recruitment of several downstream effectors and the activation of cascades of signal transduction [150,151].

Interestingly, both p75NTR and TrkB are expressed in dividing progenitor cells in the subventricular zone and SGZ of adults [152,153], BDNF and its role in neurogenesis has been studied extensively as any of the other neurotrophins, and BDNF directly impacts in an increase of the neurogenesis of granular cells in adults [154].

Similarly, direct intraventricular administration of BDNF as a result of the use of adenoviruses transfected with BDNF in the lateral ventricle resulted in a significant increase in newly formed neurons that originated in the adult SVZ [155,156]; the conditional loss of TrkB signaling in a Nestin-CreERT2 system resulted in decreased growth of neurospheres induced by BDNF in vitro, as well as damaged proliferation and neurogenesis in adult DG in vitro, suggesting that functional TrkB signaling is required for the proliferation of NSCs in the hippocampus [153]. A different study showed that survival, dendritic arborization, and functional integration of the neuron in newborns and adults depends critically on signaling through the TrkB receptor [157].

On the other hand, the improvement of hippocampal neurogenesis was not found in mice in which BDNF gene expression was silenced [158], suggesting that the role of BDNF in neurogenesis in adult SVZ [159] does not stimulate neurogenesis in the adult. For adult SVZ [159] however, the data available on the role of NT-3 in adult neurogenesis are limited in a negative response to neuronal differentiation, but not to proliferation in the adult hippocampus [160].

In addition, mice deficient in NT-3 had profound deficits in memory and learning, suggesting that neuronal differentiation mediated by NT-3, but not proliferation, is involved in spatial learning and memory formation in the adult brain [161]. The continuous infusion of NGF directly into the lateral ventricle of the adult rats had no effect on the proliferation of progenitor cells in the DG granular cell layer, but resulted in longer survival of the neurons in the adult hippocampus [161]. 

Growth factors include a large group of extracellular proteins that promote cell growth and maintenance in various biological environments [162,163], Several growth factors have been reported to be involved in neurogenesis in the adult brain, which is more important. For fibroblast growth factor-2 (FGF-2), insulin-like growth factor (IGF-1), and vascular endothelial growth factor (VEGF), these growth factors share a common principle of signal transduction, associated with a family of tyrosine kinases. Binding of the ligand to the receptor results in autophosphorylation and subsequent activation of downstream signaling pathways, including PI-3 kinase/Akt and the route Ras/Raf/-MEK/Erk. Several recent studies have implicated FGF-2 as a regulator of neurogenesis in the adult brain. Intraventricular infusion of FGF-2 was characterized by an increase in the number of newborn cells in the hippocampus of the adult rat [164].

In addition, mice with conditional deletion of the FGFR1 gene show a significant deterioration in the proliferation of neuronal progenitor cells and in the production of new neurons in adult DG [165], the role of IGF-1 in the regulation of adult neurogenesis has been addressed in several studies. Spontaneous neuronal differentiation of progenitor cells derived from adult SVZ depends on the endogenous signaling of IGF-1 in vitro [166], IGF-1 can directly stimulate the proliferation of adult hippocampal progenitor cells in vitro from a MAP kinase [167], and IGF-1 increases the rate of neurogenesis in the adult hippocampus in vivo when administered by continuous subcutaneous infusion or intraventricular infusion [168,169].

In addition, IGF-1 signaling is necessary for adequate migration of SVZ neuroblasts to the olfactory bulb through the rostral migratory current [170]; in addition to promoting adult neurogenesis, it was discovered that IGF-1 instructively stimulated the differentiation of adult hippocampal progenitor cells into oligodendrocytes in vitro and in vivo by inhibiting BMP signaling [171].

VEGF has become a multifunctional growth factor that participates in the regulation of growth and maturation of neurons during development, and can influence complex processes in the adult brain, including learning and memory [172,173]. VEGF signals through two high affinity tyrosine kinase receptors, Flk-1 and Flt-1 [152,174]. VEGF receptors are expressed in endothelial cells and neuronal progenitors in the adult hippocampus and SVZ [175]. Jin and colleagues demonstrated that VEGF exerts a mitogenic effect directly on neuronal progenitor cells through a mechanism dependent on Flk-1 [176]. The same study found that VEGF in the lateral ventricle of adult adolescents increases neurogenesis in the SVZ and SGZ [177]. VEGF-Flk1 signaling is necessary for antidepressant-mediated improvement of neurogenesis in the adult rat hippocampus [178].

## 7. Competition for the Resources of the Microenvironment

An example of the differentiation resulting from competition for a microenvironment resource is oligodendrocyte progenitor cells (OPCs) derived from the ventral ventricular zone, representing the first wave of cells of the oligodendrocyte lineage generated in the spinal, and eventually giving rise to 85–90% of the final oligodendrocyte population found in this organ cord. In this case, competition for limiting quantities of growth factors like platelet-derived growth factor (PDGF) might be a determining factor for winning the competition [179].

The differentiation of OPCs to oligodendrocytes and the onset of myelination are spatially and temporally regulated, involving signaling processes between the Notch1 receptor, its ligand Jagged 1 located on the axonal surface, and γ-secretase [180]. Interestingly, oligodendrocytes have only a brief period of time for myelination early during differentiation, and are relatively incapable of myelinating once they are mature, to fund the modulation focusing on environmental factors affecting OPC outcome during a critical temporal window—the period between final cell division and terminal oligodendrocytes differentiation [181]. 

## 8. Bone Morphogenetic Protein

Bone morphogenetic protein (BMP) comprise the largest subgroup of the superfamily of TGF-beta [182], BMPs are highly expressed in embryonic and adult nervous systems and play a fundamental role in a wide variety of cellular processes, including cell survival, proliferation, and destination specification [183]. The activities of BMPs are negatively regulated by Noggin, Chordin, and Neurogesin-1, proteins that directly bind to and antagonize BMPs extracellularly [184]. BMP signaling is transduced through two different types of receptors: serine-threonine kinase and BMP receptor type I and type II [185]. Binding of the BMP gene results in the formation of a tetramer complex of two BMP type I receptors and two BMP type II receptors, and activates an intracellular signaling cascade which involves phosphorylated Smad proteins [186]. Smad1/5/8 are directly phosphorylated and activated by the type I BMP receptor kinases, and then form a heteromeric complex with a Co-Smad, Smad4. Activated Smad complexes are translocated to the nucleus and, together with other nuclear cofactors, activate the transcription of several genes [187].

In the adult neurogenic network, BMPs promote glial differentiation and inhibition of neuronal target specification [188]. in the adult SVZ, the BMP ligands and their receptors are expressed through the population of stem cells and neural progenitors, and act as potent inhibitors of the neuronal differentiation of type B and C cells. In addition, it was discovered that BMPs are important to promote the survival of neuroblasts that migrate along the RMS [189]. Interestingly, the BMP inhibitor Noggin is produced by the SVZ and antagonizes endogenous BMP signaling and premature glial differentiation mediated by BMP at the expense of neurogenesis, which promotes the formation of new neurons from SVZ precursors, in the adult SGZ, Noggin has been produced endogenously in vitro and in vivo [190]. Noggin has also been produced endogenously for the self-renewal and proliferation of adult hippocampal NSC cells. Levels of Noggin mRNA in the dentate gyrus are under the control of the RNA binding protein FXR2. Loss-of-function experiments have shown that FXR2 results in increased expression of Noggin. The increased levels of noggin inhibit endogenous BMP signaling, which in turn results in increased proliferation of NSCs, and thereby increases neurogenesis in the adult hippocampus in vivo [93].

In addition, Neurogesin-1, a newly identified astrocyte-derived signaling protein, plays an important role in the specification of cell fate mediated by BMP in the adult brain. Neurogestin-1 is highly expressed in the adult DG and SVZ, and antagonizes astroglial differentiation induced by BMP-4 from progenitor cells of the adult hippocampus [191]. The blocking of BMP signaling by direct intraventricular infusion of Noggin as well as the knock-out of Smad4 in adult SGC neuronal precursor cells initially increased neurogenesis, but resulted in the depletion of precursors and the loss of neurogenesis, suggesting that BMP signaling is necessary for the maintenance of neural stem cell properties and neurogenesis [192].

## 9. Neurotransmitters

Neurotransmitters are small diffusible molecules that serve as a basis for chemical communication between neurons [193]. The accumulation of evidence also indicates the role of neurotransmitters in the proliferation, differentiation, and synaptic integration of adult progenitor cells, as well as in adult neurogenesis dependent on activity. Glutamate is an excitatory neurotransmitter that uses several different receptor subtypes, namely ionotropic NMDA receptors, AMPA, and kainic acid, as well as metabotropic glutamate receptors [194]. Electrophysiological and immunohistochemical studies have shown the expression of various glutamate receptors in neural progenitor cells in the subventricular adult and SGZ [195]. Interestingly, in the postnatal SVZ, the neuroblasts but not stem cells express several receptors glutamatergic during migration to the olfactory bulb [196] and neuroblast migration is detected by specialized cells such as astrocytes, to release glutamate. The silencing of the NMDA receptor results in apoptosis of neuroblast migration, suggesting that glutamate derivative astrocytes mediate survival and adequate functional integration of neuroblasts through NMDA receptor signaling [195]. The kainate GLU K5 receptor is activated in the SVZ neuroblasts that migrate, and this activation decreases the migration rate of the neuroblasts [197]. Glutamate exerts differential effects on the production and migration of neuroblasts; it has been hypothesized that the differences in the expression of the glutamate receptor between neuroblasts may be due to their state of differentiation or their final destination in the olfactory bulb [198]. Recent studies have addressed the role of glutamatergic signaling in the regulation of proliferation and the choice of fate in the adult hippocampus. Interestingly, the NMDA receptor subunits NR1 and NR2B are absent from the transient amplifying progenitors, but are found in type 1 precursor cells that express GFAP in the adult hippocampus [199]. BHLH transcription factors Hes1 and Id2, and NeuroD expression of the proneural transcription factor [200], were found in NMDA receptors in the proliferation of adult hippocampal progenitors. By using a single-cell retrovirus-mediated gene knockout technique in mice, Tashiro et al. The NMDA glutamate receptor in a short period after birth, showed that the survival of new neurons is critically regulated by neuronal activity. In addition to the critical role of glutamate signaling mediated by the NMDA receptor, emerging evidence suggests a role for cainic acid and AMPA receptors in adult hippocampal neurogenesis. 

In order to investigate the rate of neurogenesis under pathological conditions, such as epilepsy, the kainate receptor agonist kainic acid is used as an in vivo inducer of seizures. Seizures induced by Kainic acid result in a long-lasting generation of functionally integrated neurons in the adult rat hippocampus [201]. In addition, chronic administration of the AMPA receptor enhancer increased the proliferation of progenitor cells in adult DG in vivo [202].

GABA is the main inhibitory neurotransmitter in the adult brain. GABA exerts a double role in the immature cells of new granules, initially depolarizing and subsequently, hyperpolarizing, depending on the intracellular chloride content that determines the transmembrane gradient. The GABA receptor is more important for the GABA A receptor, which is an anotropic receptor channel that passes through the binding of GABA with the polarity in the chloride gradient across the membrane [203]. In postnatal SVZ, GABA released from neuroblasts reduces the proliferation rate of NSCs expressing GFAP through tonic activation of the GABA A receptor, which now provides proliferation of neural progenitor cells [203]. In addition, it was demonstrated that GABA has a direct effect on the migration of neuroblasts in adult SVZ. GABA derived from the cells surrounding the astrocytes slowed the migration of the neuroblasts on the way to the olfactory bulb through signaling mediated by the GABAA receptor (GABAAR) [204]. Ge et al. showed that newborn granular cells of the adult hippocampal DG are activated by the environment [205]. In addition, retroviral mediated expression of short hairpin RNAs against NKCC1, a Na-K-2Cl transporter, decreased the concentration of intracellular chloride, resulting in cellular hyperpolarization upon application of GABA. GABAergic and glutamatergic synapses, as well as the decrease in dendritic complexity [205], have been reported as a result of premature hyperpolarization of immature neurons. The GABA hippocampus is derived from local interneurons expressing parvalbumin, which promotes inactivity of the neuronal stem cells of the radial hemisphere hippocampus in response to neuronal activity and experience Interestingly, the conditional deactivation of the gamma 2 subunit of the GABA A receptor resulted in a rapid quiescence output and improved symmetric self-renewal, suggesting the signaling of GABA-gamma 2 as an important mechanism involved in the activation and self-renewal mode of quiescent adult NPCs [206].

Dopamine is a neurotransmitter of catecholamine that is involved in ontogenesis and embryonic proliferation of the germinal zone during development, and modulates movement, mood and motivation in the adult brain [207]. Dopamine receptors are classified as D1-like (D1 and D5) or D2-like (D2, D3 and D4), according to structural homologies and shared cascades of second messengers [208]. In adult SVZ, type D2 receptors are expressed predominantly in transient amplifying cells (type C cells), which are the target of dopaminergic afferents of the anterior brain. Interestingly, dopaminergic denervation of type C cells expressing EGFR resulted in a significant reduction in the proliferation rate of SVZ progenitor cells [209]. A subsequent study showed that the dopaminergic fibers that innervate the SVZ originate, at least in part, in the pars compacta of the substantia nigra [210]. Stimulation of D2-like receptors in type C cells expressing EGFR through chronic administration of levodopa resulted in increased proliferation of neural progenitor cells in adult SVZ [211]. In addition, systemic administration of the D2-type agonist 7-hydroxy-N, N-di-n-propyl-2-aminotetralin (7-OH-DPAT) significantly increased the proliferation of precursor cells in adult SVZ [212]. In the adult hippocampus, the dopaminergic afferents that originate in the ventral tegmental area stimulate the proliferation of neural precursors in the SGZ [211].

## 10. Conclusions

In conclusion, the great complexity of the molecular mechanism of the differentiation of intermediate neural progenitor cells is mediated by a variety of factors, whether specific to the neuronal stem cell (intrinsic factors) or the result of the microenvironment surrounding the neuronal stem cell (extrinsic factors). At present, there are no effective treatments for neurodegenerative diseases, therefore requiring the development of new therapeutic strategies. This is only possible if we achieve a better understanding of the molecular processes that determine the fate of a neural stem cell.
